# Injectable Gelatin–Farsi Gum Hydrogels Functionalized With Hydroxyapatite for Biomimetic Oral Bone Regeneration

**DOI:** 10.1155/bmri/6156533

**Published:** 2026-08-07

**Authors:** Mohammad Amin Amiri, Mozhgan Abedanzadeh, Seyyed Sajad Daneshi, Asma Najibi, Moein Zarei, Saeed Afzoon, Fatemeh Akbarizadeh, Radmehr Kazemi, Kiarash Fereidouni, Mohammad Norouzi, Seyyed Vahid Niknezhad, Fatemeh Lavaee

**Affiliations:** ^1^ Oral and Dental Disease Research Center, Shiraz University of Medical Sciences, Shiraz, Iran, sums.ac.ir; ^2^ Department of Pharmaceutical Nanotechnology, School of Pharmacy, Shiraz University of Medical Sciences, Shiraz, Iran, sums.ac.ir; ^3^ Department of Anatomy, School of Medicine, Shiraz University of Medical Sciences, Shiraz, Iran, sums.ac.ir; ^4^ Department of Chemistry and Biochemistry, Miami University, Oxford, Ohio, USA, miami.edu; ^5^ Centre for Advanced Materials and Technologies (CEZAMAT), Warsaw University of Technology, Warsaw, Poland, pw.edu.pl; ^6^ School of Dentistry, Shiraz University of Medical Sciences, Shiraz, Iran, sums.ac.ir; ^7^ Orthodontic Research Center, School of Dentistry, Shiraz University of Medical Sciences, Shiraz, Iran, sums.ac.ir; ^8^ Pharmaceutical Sciences Research Center, Shiraz University of Medical Sciences, Shiraz, Iran, sums.ac.ir; ^9^ Student Research Committee, School of Dentistry, Shiraz University of Medical Sciences, Shiraz, Iran, sums.ac.ir; ^10^ Student Committee of Medical Education Development, Education Development Center, Shiraz University of Medical Sciences, Shiraz, Iran, sums.ac.ir; ^11^ Burn and Wound Healing Research Center, Shiraz University of Medical Sciences, Shiraz, Iran, sums.ac.ir; ^12^ Oral and Dental Disease Research Center, Department of Oral and Maxillofacial Medicine, School of Dentistry, Shiraz University of Medical Sciences, Shiraz, Iran, sums.ac.ir

**Keywords:** bone regeneration, hydrogels, tissue engineering

## Abstract

**Objective:**

Bone repair requires injectable biomaterials that solidify in situ, resist infection, and provide osteoconductive reinforcement. However, it remains unknown whether a photo‐crosslinkable hydrogel can combine silver nanoparticle functionality with hydroxyapatite‐driven mechanical support for mandibular regeneration.

**Methods:**

An injectable hydrogel was synthesized from gelatin methacrylate and methacrylated Farsi gum, functionalized with silver nanoparticles and hydroxyapatite (0%, 5%, and 10% *w*/*w*). Chemistry and phase integration were verified by Fourier‐transform infrared spectroscopy, x‐ray diffraction, and proton nuclear magnetic resonance; swelling, degradation, porosity, and compression (including cyclic loading) were assessed at 5 and 12 weeks using radiography and histology; and rat mandibular healing was assessed at 5 and 12 weeks by radiography and histology.

**Results:**

Spectroscopic and diffraction analyses confirmed incorporation of all components. All formulations showed controlled swelling/degradation with porous architectures. Hydroxyapatite increased compressive performance, with 10% *w*/*w* yielding the highest strength and elastic recovery under repeated compression. In rat mandibles, the 10% hydroxyapatite hydrogel supported bone regeneration on radiographic and histologic assessment at Weeks 5 and 12.

**Conclusions:**

These data show a single injectable platform that combines antimicrobial and anti‐inflammatory functions with osteoconductive support, paving the way for minimally invasive craniofacial and orthopedic bone repair.

## 1. Introduction

Current treatments for oral and maxillofacial bone regeneration provide structural and functional repair of defect areas. However, many of these modalities require further improvements to meet the standards concerning the biological standards for effective and long‐lasting regeneration [1]. Despite the current challenges, methods based on tissue engineering offer novel treatment options in numerous medical disciplines, including bone augmentation procedures in oral and maxillofacial surgery for dental implant placement, periodontal reconstructions, and beyond [2, 3].

Although autogenous bone grafting is the current “gold standard” procedure for large defects, it presents some limitations, such as insufficient graft volume availability, donor site morbidity, or unpredictable bone resorption [4]. Allografts and xenografts are also associated with the risk of immune reactions and pathogen transmission [5, 6]. On the other hand, although alloplastic grafts can be prepared in desired amounts, their variable resorption, brittleness, and poor clinical performance in some cases limit their application in clinical practice, particularly in the treatment of large defects [7]. To address these limitations, current bone tissue engineering strategies focus on synthesizing and integrating osteoinductive and osteoconductive biomaterials [8]. These biomaterials provide sufficient volume to prevent soft tissue collapse into the intrabony defect and promote seeded cell migration, proliferation, and differentiation through biological and mechanical cues [9].

Gelatin, a modified collagen product, has been widely utilized in bone tissue engineering due to its biocompatibility and ability to support cell attachment [10]. As the primary component of the bone organic matrix, gelatin offers promising translational potential. However, its inherent limitations, including poor mechanical rigidity and uncontrollable degradation, necessitate further refinements. To address these challenges, recent advancements in the modification of gelatin have led to a novel combination called gelatin methacrylate (GelMA). This modification allows photo‐crosslinking under UV irradiation, yielding hydrogels with tunable mechanical properties, enhanced structural stability, and controlled degradation profiles [11–14]. Since GelMA alone may not possess all the necessary features for bone tissue engineering, combining it with bioceramic materials can enhance its osteogenic capacity [15]. Hydroxyapatite (HA), a popular form of bioceramics, is a favorable choice for bone tissue engineering procedures due to its close similarity to natural bone structure [16]. HA is a stable form of calcium phosphate bioceramic, comprising the main composition of human bone and teeth [16]. This biomaterial exhibits excellent biocompatibility, bioactivity, and osteoinductivity, resulting in improved adhesion, proliferation, and differentiation of osteogenic cells [17]. Proper incorporation of HA with biocompatible polymers and scaffolds, including GelMA, has shown desirable outcomes in bone regeneration [18]. For instance, it is reported that adding HA to GelMA can enhance cell proliferation and promote the formation of an osteon‐like structure, and improve bone defect regeneration while effectively mimicking the organic‐inorganic composition of natural bone tissue [19, 20].

Farsi gum (FG) is a promising polymer for hydrogel synthesis in tissue engineering. This carbohydrate‐based polymer has gained attention in the food industry for its stabilizing, emulsifying, film‐forming, and high water‐absorption properties [21–24]. Its carbohydrate‐rich composition is particularly advantageous for scaffold fabrication, as it mimics the extracellular matrix (ECM), which is also rich in carbohydrates, thereby promoting enhanced cell and protein interactions [25–29]. To further expand the applicability of FG in tissue engineering and clinical settings, the incorporation of photo‐crosslinkable groups, such as methacrylate, has been suggested to improve its structural and functional properties [28]. Furthermore, it is demonstrated that FG has good radical scavenging ability, which can be improved by adding silver nanoparticles (AgNPs). AgNPs possess great anti‐infection potential, and their combined application with FG can result in both antibacterial and anti‐inflammatory outcomes. This combination can reduce scar formation and accelerate tissue healing. Furthermore, the addition of photo‐crosslinking ability to FG enhances its potential for use in tissue engineering and clinical applications [28].

**Scheme 1:**
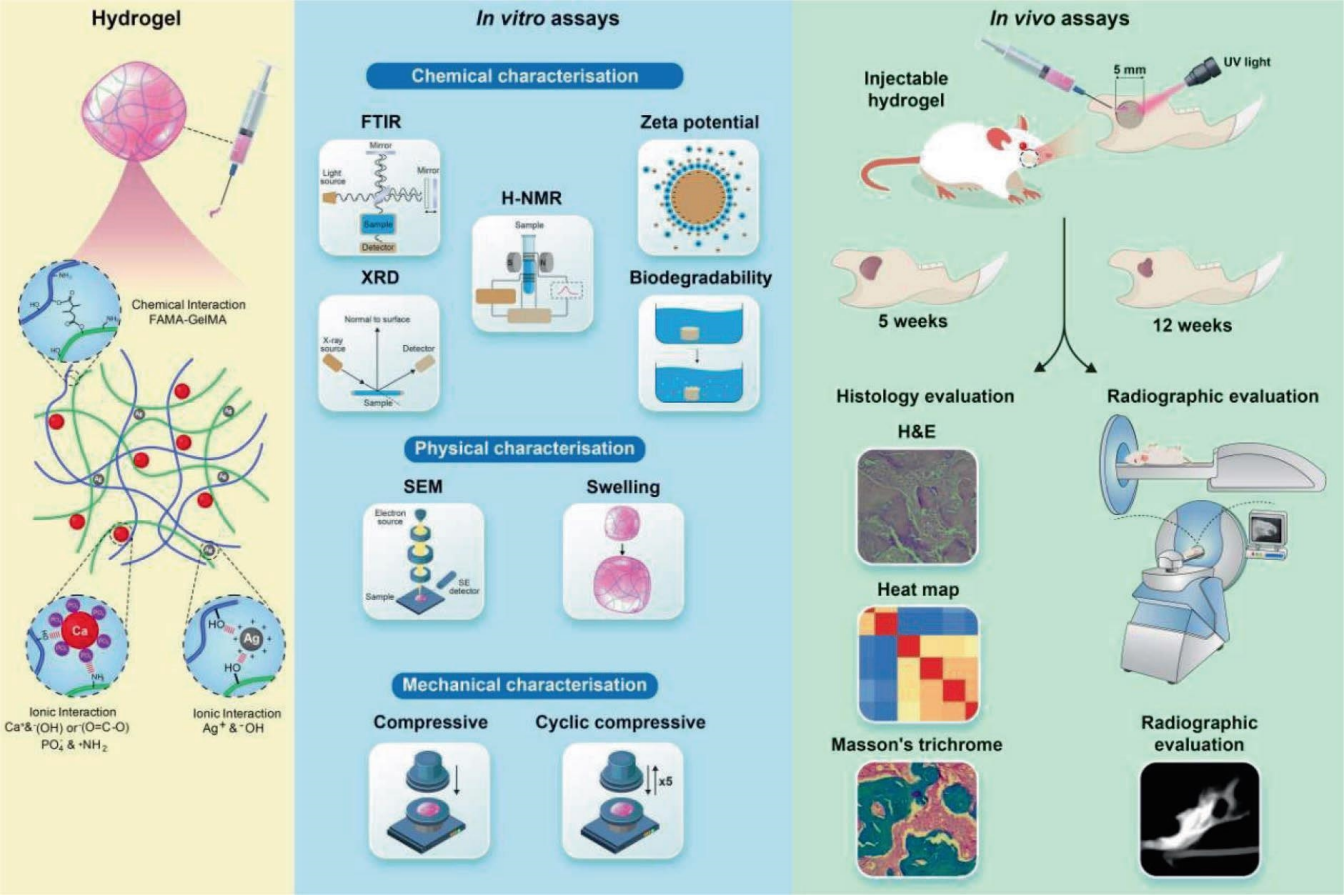
Brief schematic illustration of the present study, including hydrogel chemical structure, along with in vitro and in vivo characterizations.

Despite the achievements in the field of biomaterials for oral bone tissue engineering, current solutions still fall short of meeting the clinical demands for effective chairside applications, and further investigations are required. To bridge this gap, the authors set out to develop and characterize a novel injectable photo‐crosslinkable hydrogel using FAMA and GelMA, with enhanced handling properties and induced bone regeneration capability. The final hydrogel functionalized with AgNP and HA nanoparticles was analyzed for both in vitro and in vivo properties. Comprehensive in vitro characterization was performed, including chemical (FTIR, XRD, and ^1^H‐NMR), physical (degradation, swelling, and porosity), and mechanical (compressive strength and elastic recovery) analyses. In addition, at the in vivo stage, the mandible bone of rat models was utilized, and the rate of bone regeneration in rats was assessed on the 5th and the 12th week after surgery through histological and radiographic assessments. Final results demonstrated that the developed injectable, photo‐crosslinkable hydrogel could be a promising biomaterial for bone tissue engineering and regenerative medicine, with considerable outcomes. Scheme 1 schematically represents the different stages in the present study.

## 2. Materials and Methods

### 2.1. Hydrogel Fabrication

#### 2.1.1. Synthesis of GelMA

To synthesize GelMA, Type A porcine skin gelatin (300 g Bloom, Sigma Aldrich) was dissolved at 10% (*w*/*v*) into Dulbecco′s phosphate buffered saline (DPBS) (Gibco, Thermo Fisher Scientific, United States) at 50°C and stirred until fully dissolved. Then, 8 g of methacrylic anhydride (Sigma, United States) was added dropwise, and stirred in a dark environment for 3 h. This process was also accompanied by the addition of 200 mL of DPBS (40°C) for another 2 h. After the reaction, the mixture was dialyzed using a 10‐kDa dialysis tube (Thermo Fisher Scientific, United States) against distilled water (Merck Millipore, United States) for 7 days with repeated changes of water at 40°C, followed by lyophilization to obtain GelMA final product.

#### 2.1.2. Synthesis of FAMA‐AgNPs

To extract FG, the primary exudate of FG, obtained from a local market specializing in almond tree exudates, was dissolved in deionized water at a concentration of 5% (*w*/*v*) and incubated at 60°C for 1 h. The solution was centrifuged at 2000 rpm for 5 min. The supernatant was collected and subjected to 96% ethanol twice. The resultant extracted FG was dissolved in deionized water and freeze‐dried [30]. In order to synthesize FG‐AgNP, a primary solution of extracted FG in deionized water was prepared (1% *w*/*v*) and heated to 60°C. Then, 0.118 mmol of AgNO_3_ was added to the solution. After 24 h of strong stirring, the solution was dialyzed with SnakeSkin 3500‐Da dialysis tubing (Thermo Fisher Scientific, United States) for 48 h to remove unreacted agents. Following the synthesis of FG‐AgNP, methacrylated FG‐AgNP (FAMA‐AgNP) was synthesized. The synthesized FG‐AgNP was dissolved in 100‐mL distilled water. Then, 10 mL of methacrylate anhydrate was dropwisely added to the solution. During the process, pH was kept at 8 for 24 h in a dark environment. The final solution was dialyzed in a 10‐kDa dialysis tube for 7 days, and the water was changed twice a day.

#### 2.1.3. Fabrication of HA‐Functionalized Hydrogels

To prepare HA‐free hydrogel, GelMA and FAMA‐AgNPs, both at 5% *w*/*w* were dissolved in deionized water, followed by the addition of Irgacure 2959 (Sigma, United States) at 1% *w*/*w*, as the photo‐initiator. Then hydrogels containing HA were prepared by the addition of HA at 5% and 10% (*w*/*w*) concentrations to the previous solution. Three distinct hydrogel formulations were thus obtained: (1) control hydrogel (GelMA‐FAMA‐AgNPs), (2) low‐HA composite (GelMA‐FAMA‐AgNPs‐HA 5%), and (3) high‐HA composite (GelMA‐FAMA‐AgNPs‐HA 10%). After the complete dissolution of all components, a specific volume of each hydrogel solution was poured into predesigned molds (1‐cm diameter and 0.5‐cm height) and exposed to 365‐nm light for 5 min. All composite hydrogels underwent systematic characterization including chemical, physical, mechanical, and in vivo biological assessments.

### 2.2. Chemical Characterization Assays

#### 2.2.1. FTIR Spectroscopy

FTIR spectroscopy was used to verify the successful synthesis of GelMA, FAMA‐AgNPs, and the final composite containing HA in the 400–4000‐cm^−1^ range. The spectroscopy was performed after mixing the samples with KBr powder in a 1:100 (*w*/*w*) ratio and compressing them into tablets.

#### 2.2.2. XRD Analysis

The XRD analysis was conducted to confirm the crystalline structure and the successful synthesis of final materials using a 1.54‐Å wavelength using the D8 Advance diffractometer (Bruker, Germany).

#### 2.2.3. ^1^H‐NMR Spectroscopy

^1^H‐NMR spectra were recorded for Gelatin, GelMA, FG, and FAMA‐AgNPs, using the 400 MHz spectrometer (Avance III, Bruker, Germany) at 25°C. Samples were dissolved in D_2_O at a concentration of 10 mg/mL. The degree of methacrylation (DOM) for GelMA was calculated according to the following formula:

DOM=1100−∫lysine methylene proton of GelMA at 2.88 ppm∫lysine methylene proton of Gelatin at 2.88 ppm×.

### 2.3. Physical Characterization Assays

#### 2.3.1. Porosity, Pore Size, and Pore Area Measurement

To characterize the microstructure of each hydrogel formulation (Hydrogel, Hydrogel‐HA 5%, and Hydrogel‐HA 10%), scanning electron microscopy (SEM) (TESCAN, Vega3) was employed. Prior to imaging, each sample were photo‐crosslinked, flash‐freezed in liquid nitrogen, and cryo‐fractured to obtain optimum sample cross‐sections. Then, fractured samples were dried, and a thin layer of gold was sprayed using the spin‐coater. Porosity and pore area measurements were quantitatively analyzed using ImageJ software (Rasband, W.S., ImageJ, NIH, Bethesda, Maryland, United States) with all measurements performed in triplicate.

#### 2.3.2. Mass Swelling Assay

The effect of HA addition and its concentration (5% and 10%) on the swelling behavior and stability of the photo‐crosslinked hydrogels was characterized by assessing their hydration kinetics. The fabricated specimens were freeze‐dried. Then, the dry mass was recorded for each specimen before hydration (W_d_). Briefly, to assess the swelling behavior, hydrogel samples were immersed in DPBS and incubated at 37°C for 24 h to reach an equilibrium swelling state. At each incubation time point, the swollen weight (W_s_) of each hydrogel was recorded after gently blotting excess DPBS from the hydrogel surface with filter paper. The mass swelling ratios were then obtained from the following equation:

Mass swelling ratio %=Ws−WdWd×100.

#### 2.3.3. Degradation Assay

To evaluate the hydrogels′ degradation rate, the initial freeze‐dried weight (W_i_) of each hydrogel sample was recorded. Then, samples were incubated in DPBS, and the final dried weight (W_f_) at two time points (Days 3rd and 14th) was recorded. Fresh buffer was replaced every day. The degradation rate was calculated using the following equation:

Degradation rate %=Wf−WiWi×100.

#### 2.3.4. Zeta Potential Analysis

To assess the electrical charge of Gelatin, GelMA, FG, and FAMA‐AgNPs dispersions, at a concentration of 1 mg/mL in deionized water, ZETA‐check (Microtrac MRB, United States) was utilized. The instrument employs the oscillating displacement piston and measures the zeta‐streaming potential.

### 2.4. Mechanical Characterization Assays

Mechanical properties of each hydrogel formulation were assessed utilizing both compressive strength and cyclic compression tests (Universal Testing Machine, SANTAM, Iran). Prior to analysis, hydrogels were fabricated in cylindrical molds with 10‐mm diameter and 5‐mm height. For compressive strength test, hydrogels were compressed to fracture with a loading force of 50 N and a speed of 1 mm/min. Also, to analyze the hydrogels′ elasticity and shape recovery, cyclic compression test was performed. It involved 10 repetitions at a 1‐mm/min compressive rate to achieve a 20% strain, and finally stress–distance curves were recorded.

### 2.5. Biological Characterization Assays

#### 2.5.1. Experimental Design, Randomization, and Blinding

Twenty‐four male Sprague–Dawley rats weighing ≈200 g were included in this study. The rats were accommodated in a clean and quiet environment during the study. After general anesthesia (using intramuscular injection of ketamine/xylazine, 80/10 mg/kg), the left mandibular region was prepared prior to surgery. A 5‐mm diameter critical‐size defect (CSD) was created in the mandibular angle using a trephine bur under continuous saline irrigation. Animals were randomly assigned to experimental groups using a computer‐generated randomization sequence. The study groups were as follows (eight rats/group): control group in which defects were kept without any treatment, Hydrogel group in which defects were filled with hydrogel followed by photo‐crosslinking at 365 nm wavelength UV light, and in the last group, Hydrogel‐HA 10% was injected and photo‐crosslinked. After 5th and 12th weeks of treatment, the rats were euthanized using CO_2_ inhalation (four rats at each time point). The operated mandibular specimens were retrieved and kept in 10% formalin solution for further radiological and pathological analysis.

#### 2.5.2. Radiographic Assessment

The mandibular specimens were scanned using cone‐beam computed tomography (CBCT). The CBCT scans were performed using the NewTom NNT viewer imaging system (QR S.r.l Company, Verona, Italy) at 0.3‐mm voxel size, 110 kVp, 7.56 mAS, with a standard field of view. To precisely calculate the area of the newly regenerated bone, the empty remaining defect area was estimated and subtracted from 19.63 mm^2^ (the primarily created CSD was a circle with a diameter of 5 mm; therefore, the area was 2.5^2^ × *π* ≅ 19.63).

New bone area=2.52×π−empty remaining area.

#### 2.5.3. Histological Assessment

Operated mandibular specimens were first dehydrated using graded ethanol series (60, 70, 80, 96, and 100), then cleared in xylene, and embedded in paraffin. The paraffin‐embedded tissue blocks were sectioned at 5‐*μ*m thickness and stained with hematoxylin and eosin (H&E) (Leica Biosystems, Germany) and Masson′s trichrome (Merck, Germany), for general tissue morphology, and collagen fiber differentiation assessment. Subsequently, the areas of bone, vascular, and connective tissue, as well as the numbers of osteoclasts, osteoblasts/osteocytes, and fibroblasts/fibrocytes, were calculated using the ImageJ software (Rasband, W.S., ImageJ, NIH, Bethesda, Maryland, United States). The correlations between these factors were analyzed using R software [31].

Radiographic and histological outcome assessments were performed by investigators blinded to group allocation.

### 2.6. Statistical Analysis

Statistical analysis was carried out using SPSS software (Version 22; IBM, New York, United States). Shapiro–Wilk test was used to assess the normality of the results. Due to the normality in the distribution of the results, the ANOVA test was used to assess the differences among the groups. Besides, Tukey post hoc test was used to assess the intergroup differences. The statistically significant value was considered based on  ^∗^ *p* ≤ 0.05,  ^∗∗^ *p* ≤ 0.01,  ^∗∗∗^ *p* ≤ 0.005, and  ^∗∗∗∗^ *p* ≤ 0.001. For all the experiments, at least three replicates were performed for each hydrogel formulation.

## 3. Results and Discussion

### 3.1. Chemical Characterization

Chemical characterization of the hydrogel and its components was performed through FTIR, XRD, and ^1^H‐NMR spectroscopies.

**Figure 1:**
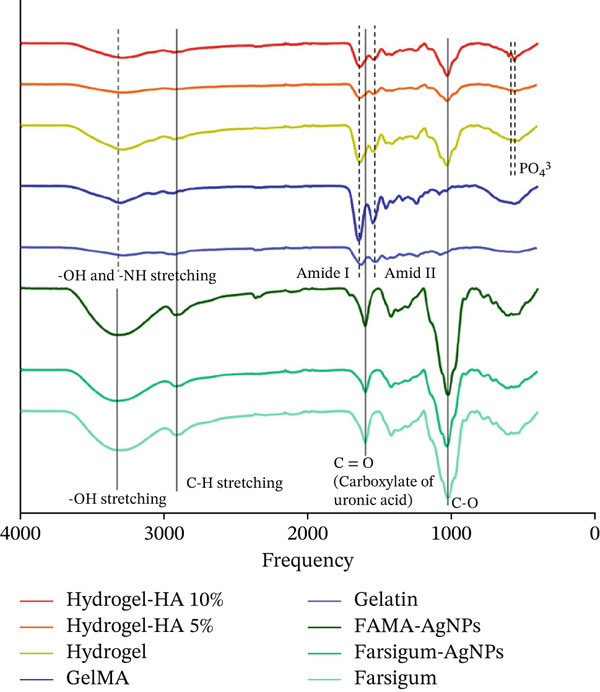
Representation of FTIR spectra of the hydrogels and their primary components.

To approve the successful synthesis of methacrylated FG (FAMA)/AgNPs, GelMA, and the final composite containing HA, FTIR analysis was carried out. Figure 1, demonstrated the FTIR spectra of the hydrogels and their primary components. Although FTIR is not a highly effective tool for differentiating gelatin and GelMA due to the overlapping of major related peaks, the enhancement of peak intensity at 1648, 1546, and 3000–3600 cm^−1^ can be related to the presence of methacrylate and the successful synthesis of GelMA [32, 33]. In addition, in gelatin and GelMA spectra, the presence of an expansive absorbance peak at 3305 cm^−1^ related to the stretching vibration of –OH and –NH, and the peaks appeared at 1646 and 1544 cm^−1^ related to amide I and amide II showed that amide bonds did not have any chemical changes after the methacrylation of gelatin [34].

The presence of the expansive absorbance peak at 3330 cm^−1^, which is related to the stretching vibration of the hydroxyl group (–OH) of FG, the absorbance peak at 2935 to 2878 cm^−1^ related to the asymmetric and symmetric stretching of CH_3_ and CH_2_ groups, the absorbance peak at 1601 cm^−1^ related to the carboxylate of FG′s uronic acid residues (C=O), the absorbance peak at 1415 cm^−1^ related to the C–O/C–H bending vibrations, and the absorbance peak at 1031 cm^−1^ related to the vibration of C–O in the hydroxyl functional group confirm the presence of FG after the methacrylation of FG and also its composition with AgNPs [35]. Upon reaction between methacrylic anhydride and FG, an ester bond was formed. The peak at 1706 cm^−1^, attributed to the C–O bond of the ester functional group, and the weak absorbance peak at 1679 cm^−1^ appeared in the FTIR spectrum of FAMA‐AgNP, which is related to C=C stretching of the alkene functional group of methacrylate modification, serving as evidence of the successful interaction between FG and methacrylic anhydride, leading to the formation of FAMA.

In the spectra of hydrogel polymer mixture, the amide I band is related to C–N and C=O stretching vibration, whereas the amide II band, related to the bending vibration of C–N and NH appeared at 1630–1645 cm^−1^ and 1540–1555 cm^−1^, respectively. Peaks in the range of 1200–1330 cm^−1^ were attributed to amide III bands of gelatin or bending vibration of the hydroxyl groups [33, 35]. The presence of HA was confirmed by the appearance of two new peaks at 561 and 599 cm^−1^ and a weak peak at 970 cm^−1^. Furthermore, the intensity of these new peaks has increased by increasing the HA percentage from 5% to 10% (Figure 1) [19].

**Figure 2:**
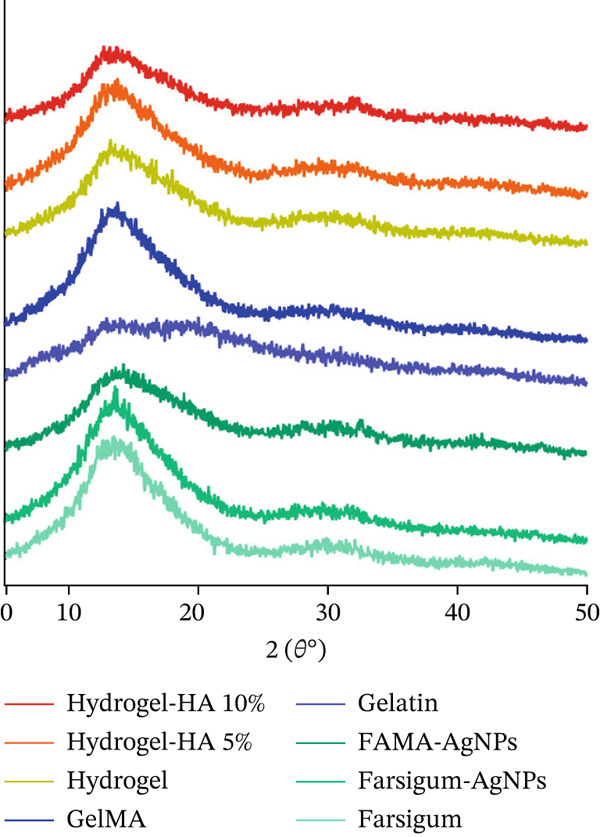
Illustration of XRD patterns of the hydrogels and their primary components.

Figure 2 and Figure S1 showed the results of x‐ray diffraction patterns for synthesized polymers and hydrogels. As it can be seen in Figure 2, the diffraction pattern of FG and pure gelatin demonstrated a broad peak over the 2*θ* range of 10°–20°, which aligns well with the previous studies and confirms the amorphous structure of FG and gelatin, with some crystalline fragments [36, 37]. According to Yakimets et al.[38] and Peña et al. [39], the distinctive peaks at gelatin diffraction pattern are usually attributed to the triple‐helical crystalline structure of gelatin. Typical x‐ray diffraction pattern of AgNP represents three diffraction peaks at 2*θ* about 38, 44, and 65 corresponding to 111, 200, and 220 planes, respectively. Figure S1b demonstrated that by the addition of the AgNP to the FG, these major peaks appeared with low intensity.

In addition, the diffraction pattern of the final hydrogels is also shown in Figure S1. The diffractograms of Hydrogel, Hydrogel‐HA 5%, and Hydrogel‐HA 10% showed a broad peak at 2*θ* = 10^°^–20^°^, confirming the presence of FG and GelMA in final hydrogels. Also, Figures S1f, and S1g demonstrated that after the addition of 5% and 10% of HA to the hydrogel, new peaks mainly at 2*θ* about 32 appeared, representing the presence of hydroxy apatite at its crystalline structure [19]. These peaks are more distinguishable in hydrogel‐HA 10%, indicating increased amount of HA.

^1^H‐NMR spectroscopy was used to investigate the chemical composition of the extracted FG, FAMA, gelatin, and GelMA (Figure S2). Several chemical shifts were observed: 3.2–4.3 ppm, corresponding to protons on the hydrocarbon rings of FG; 1.0–1.2 ppm, attributed to the methyl protons of rhamnose; and distinct peaks at 4.3 and 4.9 ppm, corresponding to the protons of glucose and galactose, respectively [28]. FG methacrylation was confirmed using ^1^H‐NMR. The appearance of two signals at 5.6 and 6.0 ppm corresponds to the vinyl protons in methacrylate groups, whereas a peak at 1.8 ppm is associated with the methyl protons of methacrylate groups, confirming successful modification [40]. The DOM of the GelMA was 29.83% according to the changes in the integration of lysine peak at 2.8 ppm. Besides, the increase in 1.8 ppm further confirmed the presence of methacrylate protons in GelMA [41].

### 3.2. Physical Characterization

**Figure 3:**
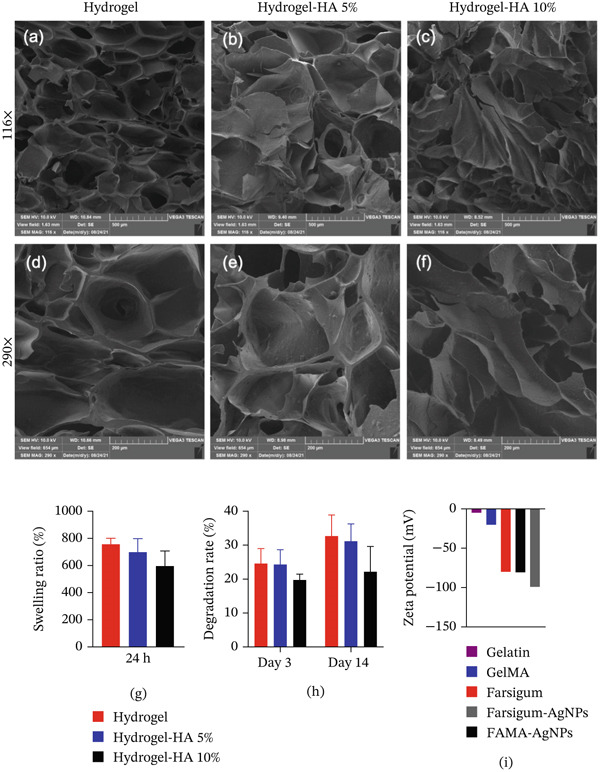
SEM images of Hydrogel (a, d), Hydrogel‐HA 5% (b, e), and Hydrogel‐HA 10% (c, f) at magnifications of 116× and 290×. (g) The swelling rates (three samples in each group), and (h) the degradation rates of Hydrogel, Hydrogel‐HA 5%, and Hydrogel‐HA 10% on Days 3 and 14 (three samples in each group), and (i) the zeta potential of basic components of the synthesized hydrogel, including Gelatin, GelMA, FG, FG‐AgNPs, FAMA‐AgNPs (three samples in each group).

Figure 3a–f showed the electron microscopic picture of internal microstructure of Hydrogel, Hydrogel‐HA 5%, and Hydrogel‐HA 10% specimens. The calculated pore size of the specimens was 311.47, 229.60, and 185.50 *μ*m for the Hydrogel, Hydrogel‐HA 5%, and Hydrogel‐HA 10% hydrogels, respectively. Also, the porosity percentage results of Hydrogel, Hydrogel‐HA 5%, and Hydrogel‐HA 10% specimens were calculated as 55.16%, 52.24%, and 51.03%, respectively. Concerning the results of pore diameter and porosity percentage of the hydrogels, it was concluded that by the addition of HA and also the increase in HA amount, both pore diameter and porosity percentage significantly decreased. This happening could be the result of increased crosslink density of the hydrogels.

As it can be seen in Figure 3g, the results indicated that the mass swelling of Hydrogel, Hydrogel‐HA 5%, and Hydrogel‐HA 10% specimens were 855.96%, 771%, and 695.50%, respectively. Also, degradation analysis showed weight loss of 24.61%, 25.71%, and 17.66% on Day 3 and 33.57%, 28.84%, and 19.01% on Day 14, for Hydrogel, Hydrogel‐HA 5%, and Hydrogel‐HA 10% specimens, respectively (Figure 3h). The outcomes indicate that the addition of HA to hydrogel can minimize degradation and swelling rates, which could be supported by a previous study performed by Zuo et al. [19] that showed the addition of 1%–3% HA to pure gelatin reduced the swelling ratio from 280% to 100% after 25 h. Similarly, Wang et al. [41] demonstrated that increasing HA concentration (0.1%–5%) in GelMA/polyethylene glycol diacrylate (PEGDA)/HA hydrogels significantly reduces pore size and swelling, which aligns with our outcomes of pore size and the swelling. The increase in HA concentration decreased the swelling rate of the GelMA/PEGDA/HA hydrogels which could be as a result of calcium part of HA interaction with the oxygens of the hydroxyl groups in the hydrogel [41].

The zeta potential analysis of FG, FG‐AgNPs, FAMA‐AgNPs, gelatin, and GelMA was assessed in a water solution with a pH of 7.23. The results indicated that FG has a zeta potential of −79.767 mV (Figure 3i). The surface charge of FG became more negative when AgNPs were incorporated (FG − Ag = −99.13 mV). However, the process of methacrylation of FG‐AgNPs reversed this trend (FAMA − AgNPs = −80.34 mV). On the other hand, the zeta potential of gelatin was estimated at −3.6 mV, whereas the zeta potential of GelMA was −20.034. The possible reason for the zeta potential of GelMA being more negative than the gelatin specimens can be attributed to the fact that based on the ^1^H‐NMR analysis, the methacrylate groups bond to the amine group of the lysine in gelatin. Therefore, the process of methacrylation signifies the negative charge of carboxyl groups by omitting the role of lysine amines in the surface charge of gelatin [42].

**Figure 4:**
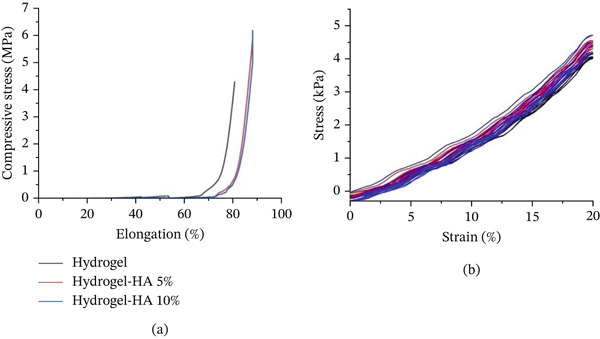
The (a) static and (b) cyclic compressive test of Hydrogel, Hydrogel‐HA 5%, and Hydrogel‐HA 10%. The cyclic compressive test illustrated in Part (b) represents five cycles of force implementation and subsequent recovery (three samples in each group).

Figure 4 presented the mechanical assessment of hydrogels. The addition of HA to the scaffolds resulted in improved static and cyclic mechanical properties, as evidenced by the increased maximum compressive stress values. The Hydrogel group exhibited maximum static compressive strength ranging from 4 to 4.5 MPa (Figure 4a). With 5% HA, the stress at break increased to 5.6 MPa, and with 10% HA, it further rose to 6.3 MPa. These results suggest that HA significantly influenced the mechanical properties of the hydrogels, possibly through reinforcing the structure. The observed increase in maximum compressive stress can be attributed to HA′s high compressive strength [43]. This bioceramic possesses high compressive strength and stiffness, contributing to enhanced load‐bearing capacity and structural integrity of the hydrogel [43]. As the concentration of HA increased from 5% to 10%, the mechanical properties improved further, indicating a dose‐dependent relationship between HA content and mechanical strength.

The interaction between HA particles and the hydrogel polymeric backbone is likely to be the other reason for this improvement. Wang et al. demonstrated that biomimetically mineralized HA enhanced the mechanical strength of the GelMA (10% *w*/*w*) by reinforcing a denser network and improving energy dissipation [20]. Similarly, Wang et al. [44] reported that the increase in HA concentration improved hydrogel compressive strength. However, in hydrogels with higher GelMA concentrations (10% or 15% *w*/*w*), excessive HA content may reverse this trend by interfering with polymer photo‐crosslinking through impeding sufficient light exposure for optimal hydrogel crosslinking. Therefore, optimizing HA content and polymer concentration proportions is critical for optimized mechanical strength.

In the cyclic compressive test, the scaffolds exhibited consistent stress–strain patterns and mechanical properties throughout the cycling process. At a strain level of 20%, the cycling stress for Hydrogel remained approximately 3.8 kPa. For Hydrogel‐HA 5%, the cycling stress was around 4.5 kPa, and also Hydrogel‐HA 10%, showed a similar increase, which was not significant between Hydrogel‐HA 5% and Hydrogel‐HA 10% hydrogels (Figure 4b). These findings indicate that HA enhances the mechanical stability of scaffolds during cyclic loading, further confirming the trend observed in static compression tests. The mechanical stability of these scaffolds under cyclic compression suggests their suitability for dynamic environments, supporting their potential for successful integration with surrounding tissues in vivo and clinical conditions. However, it is important to note that additional studies, including long‐term cyclic loading and fatigue tests, are required to further evaluate the hydrogels′ performance and assess their long‐term mechanical stability.

### 3.3. Biological Characterization

#### 3.3.1. Radiographic Assessments

To analyze the effect of HA functionalized hydrogel in bone regeneration, an animal study was performed utilizing male Sprague–Dawley rats in which the 5‐mm diameter CSD defect was induced. Study groups included: control group (defects were kept without any treatments), Hydrogel group (defects were filled with hydrogel photo‐crosslinked by 365‐nm UV light exposure), and Hydrogel‐HA 10% (filled with hydrogel‐HA 10% photo‐crosslinked by 365‐nm UV light exposure). After the 5th and 12th weeks of treatment, the mandibular specimens were retrieved for further radiological and pathological analysis.

**Figure 5:**
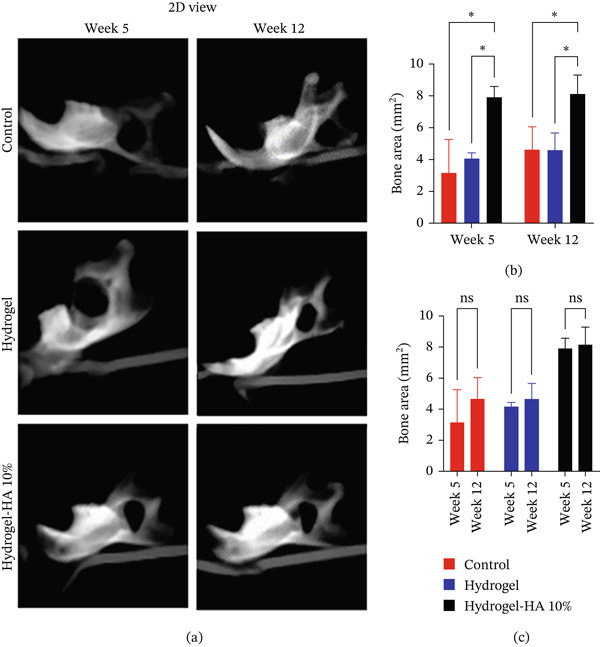
(a) The coronal 2D view of the control group, Hydrogel group, and the Hydrogel‐HA 10% group at Weeks 5 and 12, (b) comparison based on the ratio of new bone area (NBA) to the total area (TA) between the groups, and (c) between different time points (four rats in each group).

Figure 5 showed the coronal 2D view of the radiological scan of mandibular specimens at the 5th and 12th weeks of treatment for control, Hydrogel, and the Hydrogel‐HA 10% groups. As it can be seen, the Hydrogel‐HA 10% group exhibited a significantly larger area of regenerated bone when compared with the group treated with Hydrogel and the control group. Based on the outcomes concerning the comparison of different timelines, it was found that the Hydrogel‐HA 10% group had the fastest rate of bone regeneration compared with the other two groups. Despite the time‐dependent increase in the amount of regenerated bone, no statistically significant difference was found comparing the outcomes on the 5th and 12th weeks of treatment for each group. The percentage of new bone to the total defect area in Hydrogel‐HA 10% in our study was similar to the study by Hou et al. [43]. They found that GelMA (10%) with 15% and 25% HA resulted in a new bone volume comprising 40% of the total defect volume. The current study also achieved almost the same result with Hydrogel‐HA 10%. Moreover, they found that the implantation of GelMA (10%) with no HA almost exhibited the same results as the negative control which further confirms our findings [43].

In another study by Song et al. [45], the size of HA particles was emphasized as an important contributing factor in bone regeneration evaluated in CBCT radiographic analyses. They performed their analyses on GelMA with 15% *w*/*v* and 30% HA with different particle sizes, less than 10‐*μ*m particles and 50‐nm particles. They found that the calvarial defects filled with 50‐nm HA had greater regenerated bone volume on both the 4th and the 12th weeks after surgery [45]. Furthermore, Lee et al. [46], demonstrated that the sole application of osteoinductive and osteoconductive bioceramics, especially HA or *β*‐TCP, is not sufficient to achieve acceptable clinical outcomes. Their results indicated that the combined application of GelMA/*β*‐TCP or GelMA alone could result in more satisfactory outcomes compared with the sole application of *β*‐TCP. The CBCT scans indicated that *β*‐TCP particles primarily drifted out of the defect area. This poor adhesion to defect edges may have hindered osteoprogenitor cell migration and angiogenesis. This underscores the advantage of combining hydrogels—adaptable biomaterials that improve surface interaction with defect areas—with osteoinductive and osteoconductive agents such as HA [46].

#### 3.3.2. Histological Assessment

**Figure 6:**
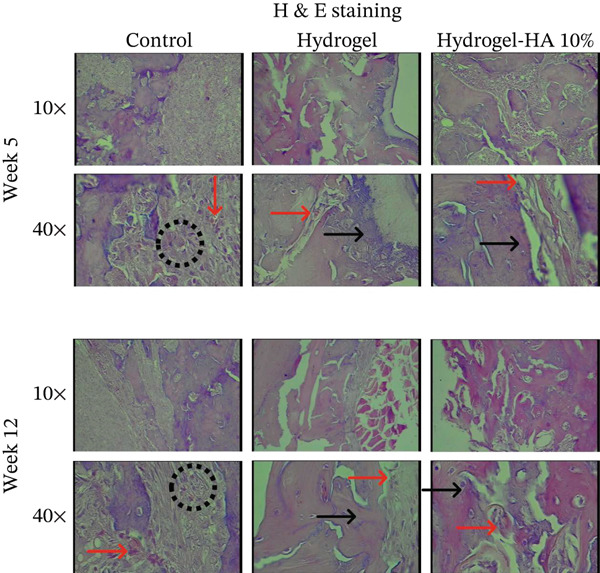
The H&E staining of the control group, the group treated with hydrogel, and the group treated with Hydrogel‐HA 10% in two time points of the 5th and the 12th week. Black dashed circle indicated connective tissue, black arrow indicated bone regenerated tissue and red arrow indicated new vessels (four rats in each group).

**Figure 7:**
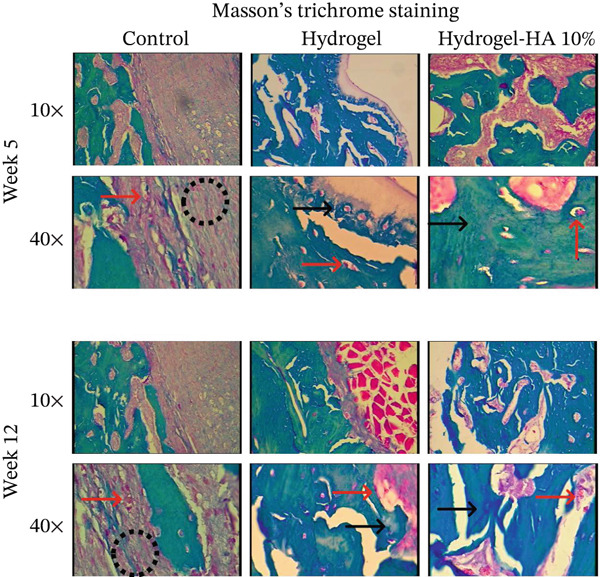
The Masson′s trichrome staining of the control group, the group treated with hydrogel, and the group treated with Hydrogel‐HA 10% in two time points of the 5th and the 12th week. Black dashed circle indicated connective tissue, black arrow indicated bone regenerated tissue, and red arrow indicated new vessels (four rats in each group).

Figures 6 and 7 showed the pathological results of H&E and Masson′s trichrome staining of the control, the Hydrogel‐treated, and the Hydrogel‐HA 10%–treated groups in two time points of 5th and the 12th week. They revealed no infiltration of inflammatory cells or foreign body reactions. In 5th and 12th weeks of treatment, the defect in the control group was prominently occupied with connective tissue (black dashed circle), whereas bone regeneration was not remarkable. The Hydrogel‐HA 10% group exhibited greater bone regeneration (black arrow) and angiogenesis (red arrow) than the control group, with reduced connective tissue formation. New bone and blood vessel formation were most noticeable in the Hydrogel‐HA 10% group on Week 12 (Figures 6 and 7).

**Figure 8:**
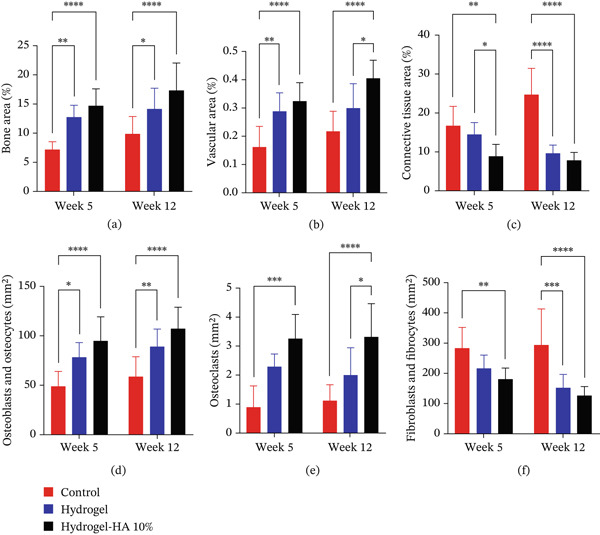
The percentage of the (a) bone area, (b) vascular area, and (c) connective tissue areas, and the distribution of (d) osteoblasts + osteocytes, (e) osteoclasts, and (f) fibroblasts for control, Hydrogel, and Hydrogel‐HA 10% at both 5th and 12th weeks of surgery (four rats in each group).

Figure 8 represented the percentage of the bone area, the vascular area, and the connective tissue areas, and the distribution of osteoblasts + osteocytes, osteoclasts, and the fibroblasts for control, Hydrogel, and Hydrogel‐HA 10% at both 5th and 12th weeks of surgery.

Regarding bone quantity, the Hydrogel‐HA 10% and Hydrogel groups outperformed the control group on Weeks 5 and 12. However, the Hydrogel‐HA 10% group showed a higher statistically significant difference (Figure 8a). A similar pattern of outcomes was observed in osteoblasts and osteocytes densities among the groups (Figure 8d). In terms of osteoclasts densities, Hydrogel‐HA 10% demonstrated significantly higher outcomes than the control group at both time points. In addition, the number of osteoclasts was higher in Hydrogel‐HA 10% compared with the Hydrogel group on Week 12. However, no significant difference in osteoclast density was observed between the Hydrogel and control groups (Figure 8e). In the study by Allen et al. [18], a 3D bioink composed of GelMA, gelatin, and HA was developed demonstrating the ability of osteoblastic differentiation, mineralization, and osteogenic gene expression. In another study by Zhang et al. [47], it was demonstrated that GelMA‐HA in both forms of hydrogel and cryogel could enhance osteogenic‐related gene expression resulting in osteogenic differentiation in calvarial defects of rats.

Concerning the vascular area, Hydrogel‐HA 10% achieved the highest outcomes at both time points (Figure 8b). Worth mentioning, both Hydrogel‐HA 10% and Hydrogel showed significantly higher rates of vascularization compared with the control group. This indicates that both the GelMA‐FAMA‐AgNPs polymers and HA significantly contribute to vascularization. The outcomes are in line with the current study performed by Im and Lin [48] that demonstrated the role of GelMA in inducing vasculogenesis through facilitating cell migration and matrix remodeling. Additionally, Chen et al. [49] have also indicated GelMA can preserve vascular network for up to 2 months under regular culture conditions. Also, Lin et al. [50] demonstrated that the subcutaneous injection of photo‐crosslinked GelMA into mice results in vascular network and functional anastomoses within 7 days. Besides, HA is demonstrated to enhance cell survival of human umbilical vein endothelial cells, lumen formation, and vascular endothelial growth factor secretion through the PI3K/Akt pathway [51].

The assessment of the connective tissue indicates the least performance for Hydrogel‐HA 10% at both time points (Figure 8c). This difference was significant when compared with the Hydrogel and the control group on Week 12 and the control group at Week 5. Additionally, the Hydrogel group showed a significant reduction in connective tissue compared with the control group at Week 12. A similar pattern was observed when assessing the densities of fibroblasts and fibrocytes, with Hydrogel‐HA 10% exhibiting the least density compared with the control group at both time points (Figure 8f). The Hydrogel group also showed lower fibroblast and fibrocyte densities than the control group at Week 12. These results imply the possible inhibitory role of HA on fibroblasts and fibrocytes. In this regard, Rakshit et al. [52] reported that HA in dermal fibroblasts medium can reduce proinflammatory cytokines secretion and collagen synthesis. Another mechanism through which HA can inhibit the fibroblasts is demonstrated in the study by Sun et al. [53]. They found that HA decreases the fibroblasts′ survival rate. Moreover, the addition of HA can decrease the transforming growth factor‐*β*1 secretion, a mediator of fibrous tissue formation [53, 54].

**Figure 9:**
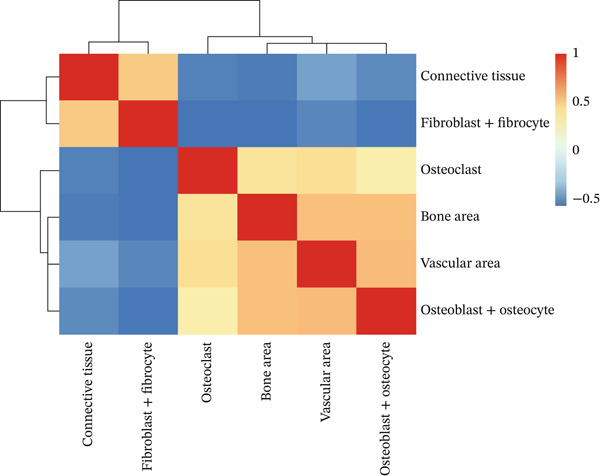
Representation of the correlation of different factors (four rats in each group) in histological analysis.

Correlation analysis of different parameters was performed between the histological variables and showed in Figure 9. The strong positive correlations among bone area, vascularization, and osteoblast/osteocyte populations suggest active bone formation supported by vascular supply, whereas the moderate link between osteoclasts and bone/vascular parameters underscores the balance between bone resorption and formation.

On the contrary, the percentage of connective tissue and the number of fibroblasts + fibrocytes showed a negative correlation with osteogenesis, angiogenesis, and the numbers of osteocyte and osteoblast.

#### 3.3.3. Limitations and Future Prospects

Despite the promising results, several limitations must be addressed. The mechanical strength of our hydrogel seems to be suitable for areas not under direct pressure. Therefore, the surgeon should be aware of the type, magnitude, and intensity of forces usually applied to the area. Computational modeling, such as finite element analysis of human bone, may provide valuable insights into the mechanical suitability of the implanted scaffolds [55]. The inclusion of fiber‐like fillers can also be a possible solution to this issue [56]. Moreover, Zhang et al. [57] suggested the addition of tannic acid to GelMA to enhance the mechanical strength through making hydrogen bonds. In addition, Wang et al. [58] stated the addition of PEGDA to GelMA can improve its mechanical strength [59]. The developed hydrogel demonstrated promising potential as a clinically applicable bone graft. From the clinical perspective, it is suitable to apply a bone graft able to flow and provide maximum contact with the defect area. This enables the clinician to crosslink the hydrogel intraoperatively. In cases where the surgeon requires a solid form of bone graft, the hydrogel can be crosslinked in molds with desired shapes before applying it inside the defect area. Additionally, it can be combined with autologous blood‐derived products, such as platelet‐rich plasma and platelet‐rich fibrin, to enhance its biological activity. This could enhance the biological activity of the hydrogel.

## 4. Conclusion

In the present study, we successfully developed an injectable, photo‐crosslinkable hydrogel composed of GelMA‐FAMA functionalized with AgNPs and HA at varying HA concentrations (5% and 10% *w*/*w*). Physicochemical characterization including FTIR, XRD, and ^1^H‐NMR analysis confirmed the successful synthesis and integration of all components, verifying chemical stability and composition. The hydrogels exhibited optimal structural properties, including a prolonged degradation rate, favorable swelling behavior, and an interconnected porous microstructure, which are critical for cell adhesion, cell proliferation, and tissue regeneration. In addition, mechanical evaluations demonstrated that HA incorporation significantly enhanced compressive strength, with the 10% HA hydrogel showing the highest mechanical robustness and excellent elastic recovery under cyclic loading. Also, in vivo studies in rat mandibular defects further validated the regenerative potential of the hydrogel, with the 10% HA group exhibiting superior bone formation, vascularization, and reduced connective tissue compared with controls, as confirmed by histological and radiographic investigations at 5 and 12 weeks postimplantation. These findings underscore the potential of Hydrogel‐HA 10% as a promising biomaterial for bone tissue engineering. Its injectability, tunable mechanical properties, and bioactivity address key challenges in regenerative medicine, particularly for craniofacial and orthopedic applications.

## Author Contributions

**M.A.A.:** writing—original draft, writing—review and editing, visualization, data curation, and formal analysis; **M.A.:** writing—original draft, writing—review and editing, and visualization; **A.N.:** writing—original draft and formal analysis; **M.Z.:** writing—original draft and formal analysis; **S.A.:** writing—original draft and formal analysis; **F.A.:** conceptualization, methodology, supervision, and validation; **R.K.:** writing—original draft and formal analysis; **K.F.:** writing—original draft and investigation; **M.N.:** writing—original draft and investigation; **S.V.N.:** conceptualization, methodology, supervision, validation, and resources; **F.L.:** project administration, conceptualization, methodology, supervision, validation, resources, and funding acquisition.

## Funding

This research was funded by a dentistry student thesis grant from Shiraz University of Medical Sciences.

## Disclosure

All authors reviewed and approved the final manuscript. The funder had no role in the study design, data collection, data analysis, interpretation of the results, manuscript writing, editing, approval, or the decision to submit the manuscript for publication.

## Ethics Statement

All animal procedures were reviewed and approved by the Ethics Committee of Shiraz University of Medical Sciences, Shiraz, Iran (Approval No. IR.SUMS.DENTAL.REC.1401.012). All procedures were performed in accordance with institutional guidelines for the care and use of laboratory animals, and the animal experiments are reported in accordance with the ARRIVE guidelines.

## Conflicts of Interest

The authors declare no conflicts of interest.

## Supporting information


**Supporting Information** Additional supporting information can be found online in the Supporting Information section. File S1 entitled “Supplementary.docx” contains additional characterization data for the materials and fabricated hydrogels used in this study. Figure S1. X‐ray diffraction patterns of (a) Farsi gum, (b) Farsi gum–AgNPs, (c) gelatin, (d) GelMA, (e) hydrogel, (f) hydrogel containing 5% hydroxyapatite, and (g) hydrogel containing 10% hydroxyapatite. The red reference lines represent the characteristic x‐ray diffraction peaks of silver, whereas the blue and green reference lines represent the characteristic peaks of hydroxyapatite. Figure S2. Representative ^1^H nuclear magnetic resonance (^1^H NMR) spectra of GelMA, gelatin, FAMA‐AgNPs, and Farsi gum.

## Data Availability

The data that support the findings of this study are available from the corresponding author upon reasonable request.
