# Supplementary material for: Injectable Gelatin–Farsi Gum Hydrogels Functionalized With Hydroxyapatite for Biomimetic Oral Bone Regeneration
Source: Biomed Res Int. 2026 Aug 7;2026:6156533. doi: 10.1155/bmri/6156533 (PMC13448513; doi:10.1155/bmri/6156533)
Supplement: Supplementary file 1 [file BMRI-2026-6156533-s001.docx]

**Figure S1.** X-ray diffraction patterns of (a) Farsi gum, (b) Farsi gum–AgNPs, (c) gelatin, (d) GelMA, (e) hydrogel, (f) hydrogel containing 5% hydroxyapatite, and (g) hydrogel containing 10% hydroxyapatite. The red reference lines represent the characteristic X-ray diffraction peaks of silver, whereas the blue and green reference lines represent the characteristic peaks of hydroxyapatite.

**Figure S2.** Representative ^1H nuclear magnetic resonance (^1H NMR) spectra of GelMA, gelatin, FAMA–AgNPs, and Farsi gum.
